# Novel Bi-allelic *PDE6C* Variant Leads to Congenital Achromatopsia

**DOI:** 10.29252/ibj.24.4.257

**Published:** 2019-12-28

**Authors:** Ata Bushehri, Davood Zare-Abdollahi, Hesam Hashemian, Ladan Safavizadeh, Jalil Effati, Hamid Reza Khorram Khorshid

**Affiliations:** 1 Genetics Research Center, University of Social Welfare and Rehabilitation Sciences, Tehran, Iran;; 2 Department of Ophthalmology, Ophthalmology Research Center, Farabi Eye Hospital, Tehran University of Medical Sciences, Tehran, Iran;; 3 Department of Opthomology, Ophthalmic Research Centre, Shahid Beheshti University (M.C.), Tehran, Iran;; 4 Meybod Genetic Research Center, Yazd, Iran

**Keywords:** Achromatopsia, PDE6C, Whole exome sequencing

## Abstract

**Background:**

The clinical phenotyping of patients with achromatopsia harboring variants in *PDE6C* has poorly been described in the literature. *PDE6C* encodes the catalytic subunit of the cone phosphodiesterase, which hydrolyzes the cGMP that proceeds with the hyperpolarization of photoreceptor cell membranes, as the final step of the phototransduction cascade.

**Methods:**

In the current study, two patients from a consanguineous family underwent full ophthalmologic examination and molecular investigations including WES. The impact of the variant on the functionality of the protein has been analyzed using *in silico* molecular modeling.

**Results:**

The patients identified with achromatopsia segregated a homozygous missense variant (c.C1775A:p.A592D) in *PDE6C* gene located on chromosome 10q23. Molecular modeling demonstrated that the variant would cause a protein conformational change and result in reduced phosphodiesterase activity.

**Conclusion:**

Our data extended the phenotypic spectrum of retinal disorders caused by *PDE6C* variants and provided new clinical and genetic information on achromatopsia.

## INTRODUCTION

Achromatopsia is a congenital visual disorder characterized by the absence of color discrimination, stationary visual impairment, nystagmus, photoaversion, and eccentric fixation^[^^1^^]^. It is a recessively inherited cone dystrophy demonstrable in electroretinogram phototopic responses, with the prevalence rate of 1 in 30,000^[^^2^^]^. In terms of fundus appearance, normal ophthalmoscopy is mostly expected, although lightly pigmented fundi, minimal granularity of the macula, and less frequently macular defects have been reported^[^^3^^]^. 

Potential pathogenic variants in the cone-specific *PDE6C* gene are associated with achromatopsia. *PDE6C* (ACHM5, MIM 600827) encodes the catalytic α subunit of cone phosphodiesterase^[^^4^^]^. Upon phototransduction initiation, transducin activates PDE6C through retracting its inhibitory Pγ. Hydrolysis of the cGMP by the active PDE6C results in the closure of the cone outer segment cation channels. It then proceeds with the hyperpolarization of photoreceptor cell membranes, as the final step of the cascade^[^^5^^]^. 

Variants in *PDE6C* are an uncommon cause of achromatopsia, comprising less than 2% of the cases, and their associated phenotypes have poorly been described in the literature^[^^6^^]^. The present study reports the comprehensive clinical specifications of two siblings with achromatopsia while harboring the homozygous likely pathogenic variant in *PDE6C*.

## MATERIALS AND METHODS

**Patients**

Here, we report a pedigree of our cohort for 63 families with inherited retinal degenerations. After obtaining the precise family history, the patients, a sister and a brother, were examined by an ophthalmologist for confirming the diagnosis of the retinal pathology through extensive ophthalmic workup, including detailed funduscopy, OCT, retinal FA, and electrotretinography. 

Molecular genetic investigation

Following obtaining 5-ml peripheral blood samples from all family members, genomic DNA was extracted using the salting-out method. Considering enormous genetic heterogeneity of the suggested diagnosis, cone-rod dystrophy, the proband was analyzed by WES technique. Unbiased enrichment of all genomic coding sequences was performed using SureSelect Human All Exon V6 (Agilent Technologies Inc., USA). The enriched libraries were sequenced on the Illumina Hiseq 4000™ platform (Illumina Inc., San Diego, USA). Then sequence reads in FASTQ format were aligned to reference genome GRCh38 (https://www. ncbi.nlm.nih.gov/grc/human/data) using conventional alignment tools (ELAND, BWA). Variants were called and filtered employing quality control criteria to create genome Variant Call Formatted file. Variants were then annotated using ANNOVAR database (http://www.openbioinformatics. org) relying on Ensembl gene and transcript definitions. Variant prioritization was performed based on population frequency, effect or nature of the variants, and zygosity. Annotated variants were checked against 1000 genomes (http://browser.1000 genomes.org), ExAC (http://exac.broadinstitute.org/), gnomAD, Iranome (http://iranome.ir/), and in-house exome data from 200 ethnically matched, unaffected individuals. The variants were also investigated in RetNet (https://sph.uth.edu/retnet/), HGMD® (Human Gene Mutation Database; http://hgmd.cf.ac.uk/), ClinVar (https://www.ncbi.nlm.nih.gov/clinvar), GeneReviews® (https://www.ncbi.nlm.nih.gov/books/ NBK1116/), and OMIM® (https://www.omim.org/).

The potential pathogenicity of missense variants was assessed using online prediction software tools, namely Condel (CONsensus DELeteriousness score of missense mutations) based on PolyPhen-2 (https://genetics.bwh.harvard.edu/pph2/) and SIFT (https://sift.jcvi.org/), CADD (Combined Annotation Dependent Depletion; https://cadd.gs.washington.edu/ info/), the Mutation Taster (https://www.mutation taster.org/), and the HOPE Server (https://cmbi.ru.nl/ hope/). Moreover, the conservation of the residues was assessed using GERP++ (Genomic Evolutionary Rate Profiling rejected substitutions score; https://mendel. stanford.edu/SidowLab/downloads/gerp/) and the PhastCons 100way vertebrate score (https:// hgdownload.cse.ucsc.edu/goldenpath/hg19/phastCons100way/).

After gene assessment and variant classification according to the American College of Medical Genetics and Genomics guidance [7], integration of results with patients’ phenotype was implemented for providing a clinically relevant interpretation of the findings. Concerning the validity of findings, confirmation of candidate variants and segregation analysis were performed on the two subjects and their parents using the Sanger sequencing method. 

**Molecular modeling**

To understand the possible consequences of the amino acid substitution on the three-dimensional structure of the PDE6C, the residue sequence of the metal-dependent motif of the enzyme catalytic domain was extracted from UniProtKB database through Pfam Database (http://pfam.xfam.org/protein/P51160/). Using Swiss-Model protein homology modeling database (http://swissmodel.expasy.org/interactive/ SgxfJr/models/), the structures of helices H4, H5, H12, H15, H-loop, and M-loop of PDE6C catalytic domain for the wild-type and mutant protein were generated by the application of PDE5/6cd molecule (PDB file: 3JWQ) as the template^[8]^. Other regions and loops were deleted and incrementally rebuilt into the electron density by manual editing. Chimera tool was used for the visualization and analysis of the modeled protein structure^[9]^.

**Ethical statement**

 The above-mentioned sampling protocols were approved by the Research Ethics Committee of University of Social Welfare and Rehabilitation, Tehran, Iran (ethical code: IR.USWR.REC1395761). Written informed consents were provided by the patients and their parents.

**Fig. 1:**
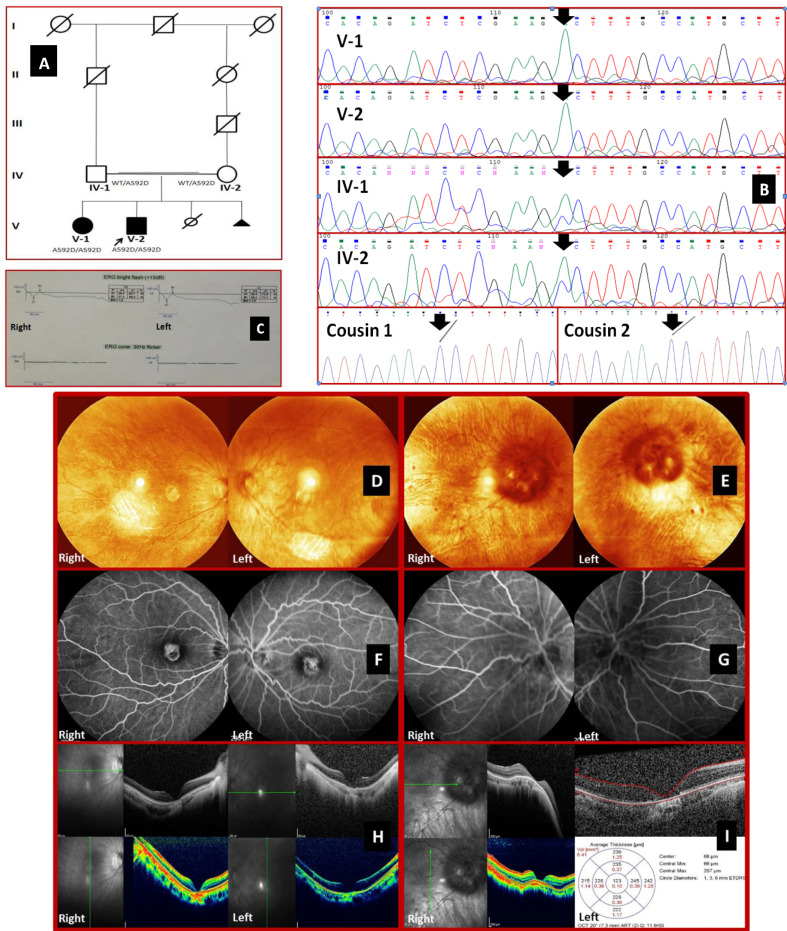
Pedigree of the family, electroretinography, sequencing electropherograms of PDE6C-related achromatopsia, fundus images of the affected siblings who have complete achromatopsia at the third decade of life. (A) Pedigree chart of the family with PDE6C variant, p.A592D, and segregation analysis of the core family. (B) Results of the electropherograms of Sanger sequencing, identifying the missense variant (c. C1775A), from family membersare compatible with their phenotypes. The parents harbored the heterozygote alleles, while two cousins had wild-type alleles. (C) Electroretinogram of the proband (V-2); the photopic response (the 30-Hz flicker) represents that cone function is absent, while the scotopic response is moderately reduced, meaning subnormal rod function. (D-E) Fundus photographs of the brother (D) and the sister (E). (F-G) FA images of the male (F) and female patients (G). (H-I) OCT of the proband (H) and his affected sib (I).

**Table 1:** Clinical description of the patients

**Subject ID/Gender. ** **age at last review**		**V-1/F.30**		**V-2/M.28**
Symptom (onset)		Nystagmus (congenital); central scotoma; photophobia; phtopsia; eccentric fixation		Nystagmus (congenital); central scotoma; photophobia; phtopsia; eccentric fixation
				
Disease progression		Stationary		Stationary
				
Best corrected visual acuity		OD: CF 2’; OS: CF1’		OD: CF 1’; OS: CF1’
				
Visual field		Small central scotoma (5°)		Central scotoma (5°-10°)
				
Fundoscopy		Atrophic macular lesion; peripapillary atrophy/drusen; signs of pathologic myopia		Atrophic macular lesion
				
OCT		SD-OCD: stage 5 (complete RPE disruption and ONL loss); RNFL OCT: thickness falls out of the normal limit in all quadrants except nasal		SD-OCD: stage 5 (complete RPE disruption and ONL loss); RNFL OCT: thickness falls out of the normal limit in all quadrants
				
Color vision		Ishihara: strong deutan; Panel D15: total achromat		Ishihara: extreme deutan; Panel D15: total achromat
				
Refractive errors		OD: -8.5; OS: -8		OD: -7.5; OS: -7.5
				
FA imaging		Missing normal foveal darkness; vascular tortuosity; peripapillary scar tissue		Central macular window defect; vascular tortuosity; optic nerve head autofluorescence

CF, counting finger; OS, left eye; OD, right eye; RNFL OCT, retinal nerve fiber layer OCT

## RESULTS

**Clinical evaluation**

A brother and a sister aged 28 and 30 at last visit, respectively were descent from first cousin once removed healthy parents (Fig. 1A). The clinical evaluations of the patients are summarized in Table 1. Also, their paraclinic evaluations are indicated in Figure 1C-I in which ophthalmoscopy of the male patient denoted near normal peripheral fundoscopic appearance, although a region of large Lacquer cracks inferior to the macula concordant to pathologic myopia could be observed. Despite similar macular changes in the fundoscopy of the female patient, it disclosed much more findings, including large optic disc, tilted optic nerve with peripapillary atrophy, peripapillary crescent and drusen, tigroid-blonde fundus, Lacquer cracks, and Dalen-Fuchs nodules, all resembling bilateral pathologic myopia. The 30-hz flicker response in electroretinogram revealed non-recordable cone function with subnormal rod function. Furthermore, SD-OCT indicated that the index patients were categorized into stage V, according to the SD-OCT features and staging system categorized by Greenberg *et al.*^[^^10^^]^, defined as complete RPE disruption and loss of the ONL with choroidal hyperreflectance. Moreover, the FA images demonstrated a transmission, or window defect, due to missing RPE that normally blocks fluorescence from choroid. As a result, the bright choroidal fluorescence evidenced the complete RPE disruption. The parents reported no visual disturbance, and also they demonstrated to be normal when undergoing a comprehensive ophthalmic examination.

***PDE6C*** **screening**

Using bioinformatics filtering strategies, a novel homozygous missense variant in *PDE6C* (NM_006204:chr10:g.95400714C>A:c.1775C>A:p.A592D) was identified in the proband. Co-segregation analysis confirmed the result (Fig. 1B). The variant, p.A592D, initially was classified as a variant of uncertain significance based on the American College of Medical Genetics and Genomics guidelines. However, there were some indicators of the pathogenicity of the variant as follows: (a) located in a critical functional domain (metal-dependent motif of the enzyme active site), (b) representing ultra-rare allele being absent in general population alleles, (c) predicted to be deleterious and evolutionary conserved, and (d) co-segregated with the achromatopsia phenotype in the family. Apart from heterozygous parents, two cousins were also analyzed as the patients without unaffected sibs. On top of that, considering the complete analysis of 269 genes registered in the RetNet database, specifically focusing on cone-rod dystrophy relevant genes and no opposing arguments regarding pathogenicity, its causality appeared to be pertinent. 

**Fig. 2:**
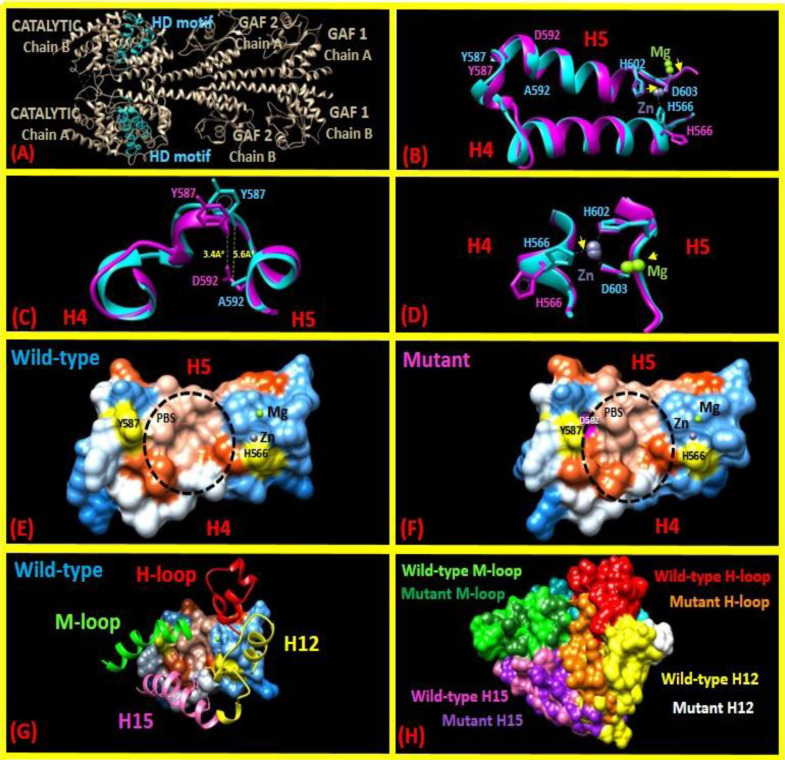
Atomic model of a conformational change in the structure of PDE6C caused by the p.A592D variant. (A) A general view of the localization of highly conserved HD motif in the PDE6C dimer protein. (B) In the model, the residue conformation and metal ion location in the catalytic site are changed by the p.A592D variant. Two alpha-helices formed by residues 562–581 (H4, bottom), 587–606 (H5, top), and 582-586 (their connecting chain) are associated with the changes caused by the p.A592D variant. (C) Local conformation changes in the vicinity of residue Y587 are caused by the p.A592D variant, demonstrating a decrease in the interatomic distance between side chains of Y587 and residue 592 from 5.6A° (A592 in the wild-type variant) to 3.4A° (D592 in the mutant variant). (D) The movement of the same Zn2+ atom is shown by cyan and magenta spheres for the PDE6C catalytic domain and for the same area of the p.A591D mutant variant, respectively. Conformations of side chains of residues H566, H602, and D603 forming a binding site for a divalent Zn2+ cation are shown. The mentioned subunits of PDE6C structure and the superimposed p.A592D mutant variant are shown in cyan and magenta, respectively. (E and F) The hydrophobic surface of PDE6C HD motif and Pγ binding site (PBS) conformational changes caused by p.A592D variant. Buried alanine at the position of 592 cannot be observed in the wild-type protein due to its hydrophobic properties, while D592 in the mutant protein would change the conformation of H5 and, consequently, H12 leading to interference in docking Pγ subunit in the PDE6C active-site pocket. (G) Interactions between wild-type HD motif and α-helices of H-loop, H12, M-loop, and H15 that make Pγ docking pocket. (H) Comparison of wild-type and p.A592D mutant active sites of PDE6C catalytic domain, which is about to be blocked by Pγ. Clearly, P.A592D variant would change conformation of Pγ docking site

**Protein structure analysis**

The *PDE6C* metal binding motif sequence showed 53.3% sequence identity to PDE5/6cd as a structural template to model residues 562-606 of *PDE6C* (Fig. 2). The p.A592D variant converts alanine (A) with hydrophobic and aliphatic moiety to aspartic acid (D) with acidic side chain that differs in size and electric charge.

## DISCUSSION

This report describes two affected siblings with achromatopsia and a novel likely pathogenic *PDE6C* variant in the family. The product of this gene consists

of a couple of cGMP-specific phosphodiesterases, adenylyl cyclases, and FhlA domains, followed by a catalytic domain. The p.A592D variant was located within the highly conserved HD motif with a predicted metal cation (Zn^2+^ and Mg^2+^)-dependent phosphor-hydrolase activity at the catalytic domain with a pivotal role in signal transduction, according to SMART protein domain annotation resource^[^^11^^]^. The A592 residue was fully conserved among vertebrates. Thus, it is assumed that the substitution introduces a negative charge in buried alanine, which probably would not fit in the core of the protein and might lead to conformation collision and folding defects^[^^12^^]^. Intriguingly, the conformation change could abrogate Zn^2+^ binding, thus potentially preventing the enzyme catalytic activity. Functional analysis of molecular modeling regarding an amino acid alteration just before A592 (E591K) illustrated that it likely decreases metal cation attachment^[^^13^^]^. On the other hand, the outcome of this modeling analysis was concordant with the results of a structural study of phosphodiesterase inhibition by the C-terminal region of the γ subunit. It demonstrated that Pγ docking site is juxta-positioned to H-loop, H12, M-loop, and H15, residues 609-632, 674-690, 747-771, and 772-793, respectively^[^^8^^]^. Our molecular modeling showed that the conformational change in H5 would interfere with Pγ functionality (Fig. 2G-H). It has also been indicated that obliteration of Pγ in mouse photoreceptors significantly reduces PDE6C hydrolyses and leads to retinal degeneration, despite the expectation that the removal of the inhibitory Pγ subunit from holoPDE6C would activate the enzyme^[^^14^^]^. It has been hypothesized that once the catalytic pockets were concealed by the Pγ subunits, aryl hydrocarbon receptor-interacting protein Like 1, as an obligate chaperone of PDE6C, would change the folded structure into "open" conformation^[^^8^^,^^12^^]^. Therefore, it could be concluded that the A591D variant might interfere with the activity of PDE6C phosphodiesterase.

Loss of function mutations of *PDE6C* decreases intracellular cGMP hydrolysis. Increased level of cGMP primarily causes the excessive opening of cGMP-gated channels in cone outer segment, resulting in an unrestricted influx of Ca^2+^. It has been demonstrated that uncontrolled cytoplasmic elevation of cGMP and Ca^2+^ cations contributes to the demise of cones^[^^15^^]^. Therefore, loss of function of PDE6C will give rise to achromatopsia.

There are quite limited published data on *PDE6C*-related retinal phenotype^[^^6^^,^^16^^,^^17^^]^. The HGMD has currently listed 38 variants in *PDE6C* that explain the disease phenotype in patients. Our cases presented with the typical features of achromatopsia since birth. Apart from pathologic myopia, fundus examination revealed atrophic chorioretinal macular corresponded to an area of complete loss of autofluorescence on FA imaging. FA signal with window defect highlights the complete RPE disruption, in keeping with stages five of the OCT classification proposed by Greenberg *et al.*^ [^^10^^]^ recently that shows severe macular thinning of all layers and excavation in retina and choroid of both eyes. Electroretinogram also confirmed complete degeneration of cones. However, the scotopic response was also concordant with subnormal rod function. Although *PDE6C* is exclusively expressed in cones, this photoreceptor-specific defect would lead to a series of cellular changes, ultimately affecting the normal function of the whole retina^[^^18^^]^. 

The novel PDE6C variant (p.A592D) in a homozygous state could explain a part of the phenotypes in our cases with achromatopsia. However, it is insufficient to explain the fundoscopic differences between the two patients. It could be explained that there might be other potentially relevant variants involved in the female index. As the WES technology was only performed in the male proband, detection of other pathogenic variants causing severe myopia was inapplicable. 

In this study, we identified a novel PDE6C variant in two cases of achromatopsia with macular atrophy. Our data extended the phenotypic spectrum of retinal disorders caused by PDE6C variants and provided new clinical and genetic information.
